# *Nigella sativa* (Black Cumin) Seed Extract Alleviates Symptoms of Allergic Diarrhea in Mice, Involving Opioid Receptors

**DOI:** 10.1371/journal.pone.0039841

**Published:** 2012-06-29

**Authors:** Swantje C. Duncker, David Philippe, Christine Martin-Paschoud, Mireille Moser, Annick Mercenier, Sophie Nutten

**Affiliations:** Nestlé Research Center, Nestec Ltd., Vers-chez-les-Blancs, Lausanne, Switzerland; INRA, UR1282, France

## Abstract

The incidence of food hypersensitivity and food allergies is on the rise and new treatment approaches are needed. We investigated whether *N. sativa*, one of its components, thymoquinone, or synthetic opioid receptor (OR)-agonists can alleviate food allergy. Hence, ovalbumin (OVA) -sensitized *BALB/c*-mice were pre-treated either with a hexanic *N. sativa* seed extract, thymoquinone, kappa- (U50'4889) or mu-OR-agonists (DAMGO) and subsequently challenged intra-gastrically with OVA. All 4 treatments significantly decreased clinical scores of OVA-induced diarrhea. *N. sativa* seed extract, thymoquinone, and U50'488 also decreased intestinal mast cell numbers and plasma mouse mast cell protease-1 (MMCP-1). DAMGO, in contrast, had no effect on mast cell parameters but decreased IFNγ, IL-4, IL-5, and IL-10 concentration after *ex vivo* re-stimulation of mesenteric lymphocytes. The effects on allergy symptoms were reversible by OR-antagonist pre-treatment, whereas most of the effects on immunological parameter were not. We demonstrate that *N. sativa* seed extract significantly improves symptoms and immune parameters in murine OVA-induced allergic diarrhea; this effect is at least partially mediated by thymoquinone. ORs may also be involved and could be a new target for intestinal allergy symptom alleviation. *N. sativa* seed extract seems to be a promising candidate for nutritional interventions in humans with food allergy.

## Introduction

The last few decades have seen a constant increase in allergic manifestations with a striking prevalence in Western Countries [1], [2]. In infants and children, food proteins are among the most potent inducers of allergic reactions. Depending on the country, the prevalence of diagnosed food allergies (double-blinded, placebo-controlled, food challenge confirmed) in the general population is between 2.4% and 4% [3]–[5] and it can increase up to 6% in children under 3 years of age [6]–[8]. Sensitization to food antigens often results in intestinal symptoms such as oral allergy syndrome, vomiting and diarrhea [9]. However, allergic reactions to food are not limited to symptoms in the gastrointestinal tract but can involve other body sites such as the skin in atopic eczema. Food allergies are often caused by different allergens according to the age of the patient. The most prevalent allergenic foods in infants and children are milk and egg [3]. Most children have outgrown their food allergies when reaching school age [10], but in some of them, symptoms persist and, moreover, they often develop allergic symptoms against other types of food such as peanut, nuts, fish, soybeans or cereals [3]. Food related allergic reactions represent a significant burden for patients and their families and cause considerable health care costs. In most of these patients, avoidance of the allergen is the only efficient treatment. This bares the risk of nutritional deficiencies, especially in children and almost always results in impairment of quality of life for the patients as well as their families. Therefore, alternative treatments are needed. As one way to find solutions research groups have conducted clinical trials with remedies from complementary and alternative medicine [11] like *Tylophora indica* in asthma [12], butterbur in allergic rhinitis [13] and St. John's wort in atopic dermatitis [14]. Another plant that has gained more and more interest for potential medical use in recent years is *Nigella sativa* (*N. sativa*). *N. sativa*, a member of the *Ranonuculaceae* family, also known as black cumin or black caraway has been shown to be rich in polyunsaturated fatty acids [15], phospholipids [16] and contains a number of active ingredients like thymoquinone, hydro-thymoquinone and nigellone [17], [18]. *N. sativa* is used in the Middle East as remedy for a number of different diseases [19], [20]. Various biological effects have been linked to the use of *N. sativa* and some of its active ingredients. In particular thymoquinone has been reported to have anti–inflammatory [21]–[23] and anti-cancer [24] properties and to alleviate allergic asthma [25]–[27]. Additionally, *N. sativa* and its component thymoquinone have been linked to anticonvulsive effects and pain protection, possibly through opioid receptor (OR) mediated mechanisms [28]–[30].

ORs belong to the G protein-coupled receptor superfamily. According to their pharmacological activity they are defined into three classes, mu-OR (MOR), kappa-OR (KOR), and delta-OR (DOR). They have first been described in the central nervous system for their involvement in pain regulation but have now been found to be widely expressed in peripheral tissues including the gastrointestinal tract. Intestinal ORs are involved in regulation of motility [31], intestinal secretion [32] and bowel transit time [33], these are mechanisms also involved in development of intestinal symptoms of food allergy. Recently, molecular and functional investigations have revealed that all three OR-subtypes are also expressed on immune cells, where they have been shown to modulate cytokine and chemokine production as well as the expression of their receptors [34].

In the present study we investigated the effect of oral hexanic *N. sativa* seed extract in ovalbumin (OVA)-induced allergic diarrhea in mice. In this well described food allergy model [35], OVA-sensitized mice develop transient diarrhea with progressively increasing severity following multiple OVA-challenges. Clinical symptoms are accompanied by elevated plasma mouse mast cell protease-1 (MMCP-1, a marker for mast cell degranulation), mastocytosis in the jejunum, elevated plasma IgE, and Th2-type intestinal responses. We demonstrated that *N. sativa* seed extract significantly improves symptoms and immune related markers in murine OVA-induced allergic diarrhea and that the effect is partially mediated by thymoquinone involving ORs.

## Results

### *N. sativa* seed extract alleviates symptoms and modulates immune markers in mice with OVA-induced allergic diarrhea

**Figure 1:**
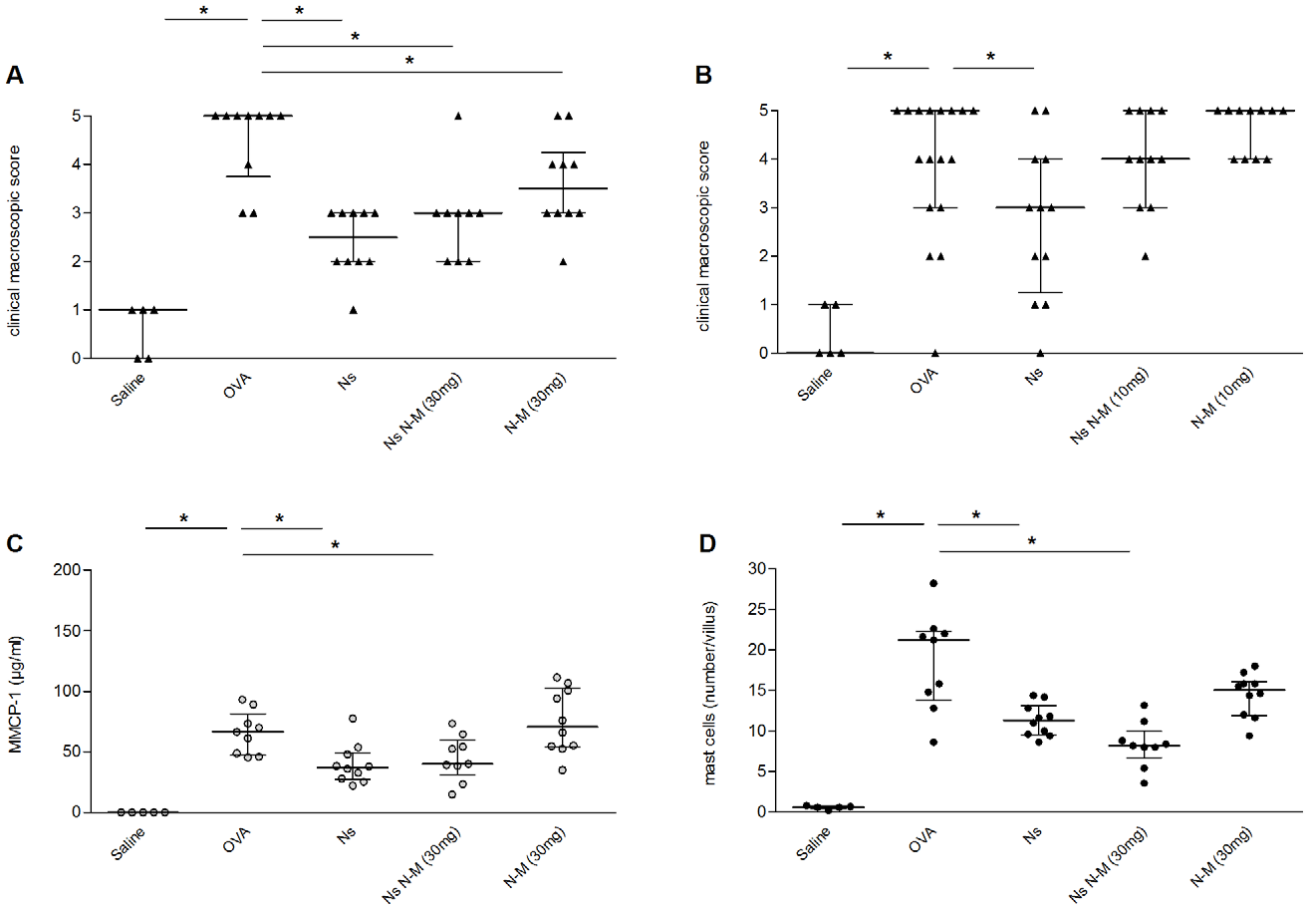
*N. sativa* seed extract decreases clinical macroscopic scores and immune parameters in OVA-allergic mice. OVA-sensitized mice were challenged with saline (Saline), OVA (OVA), or with OVA after intragastric administration of *N. sativa* seed extract (Ns) with the OR antagonist naloxone-methiodide pre-treatment at the dose indicated (Ns N-M), or with naloxone-methiodide alone (N-M). Panels **A** and **B** represent the median of clinical macroscopic scores at sacrifice. Panels **C** and **D** show the plasma concentration of MMCP-1 (**C**) and the numbers of mast cells per intestinal villus (**D**) at sacrifice. Each dot represents the corresponding value for one animal and the bars represent the median with interquartile range, * = *p*<0.05; n = 5–19.

*N. sativa* seed extract significantly attenuated the diarrhea severity (clinical macroscopic scores) at the final OVA-challenge (*p*<0.001, **Figure 1A** **+B**) as well as the sum of clinical scores from consecutive challenges (*p*<0.001, data not shown) when compared to non-treated OVA-challenged controls. The decrease in clinical symptoms was accompanied by a decrease in plasma concentration of MMCP-1 (*p* = 0.006, **Figure 1C**) and intestinal mast cell numbers (*p* = 0.005, **Figure 1D**) in mice supplemented with *N. sativa* seed extract. Total IgE or OVA-specific IgE concentrations were not significantly decreased. *N. sativa* seed extract administration did not cause any change in IL-4, IL-5, IL-10 or IFNγ release from mesenteric lymphocytes following *ex vivo* re-stimulation with OVA (data not shown).

### Thymoquinone, one of the active components of *N. sativa* seeds, alleviates symptoms and decreases immune markers in mice with OVA-induced allergic diarrhea

**Figure 2:**
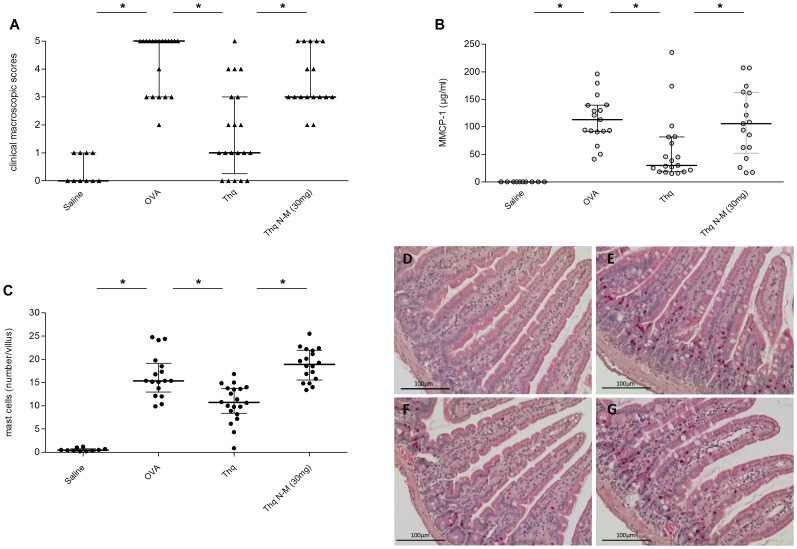
Thymoquinone decreases clinical symptoms, plasma MMCP-1 levels, and mast cell numbers in OVA-allergic mice. OVA-sensitized mice were challenged with saline (Saline), OVA (OVA), or with OVA after intragastric treatment with 13 mg/kgBW thymoquinone (Thq) and with thymoquinone after 30 mg/kgBW naloxone-methiodide pre-treatment (Thq N-M). Panel **A** represent the median of clinical macroscopic scores at sacrifice. Panel **B** shows the plasma concentrations of MMCP-1 and Panel **C** the numbers of mast cells per intestinal villus at sacrifice. The dots correspond to results from individual animals and the bars represent the median with interquartile range, * = p<0.05, panels show the results from two pooled experiments, n = 10–20. Panel **D–G** are representative histological pictures of mast cells in the jejunum of OVA-sensitized mice challenged with saline (**D**), OVA (**E**), or with OVA after intragastric treatment with 13 mg/kgBW thymoquinone (**F**) and with thymoquinone after 30 mg/kgBW naloxone-methiodide pre-treatment (**G**). Mast cells appear in bright red.

One of the most potent components of *N. sativa*, shown to modulate immune reactions, is the quinolone, thymoquinone [21], [36]. To investigate if thymoquinone was involved in mediating the positive effects of *N. sativa*, mice were treated intragastrically with thymoquinone, in a concentration corresponding to the one that had been given with *N. sativa* seed extract in previous experiments (13 µg/kg BW). Thymoquinone treatment resulted in a decrease of the sum of clinical scores (*p* = 0.006, **Figure 2A**, two pooled experiments) and the diarrhea severity at the final challenge (*p* = 0.001, data not shown). Treatment with thymoquinone also decreased plasma MMCP-1 concentration (*p* = 0.01, **Figure 2B**) and intestinal mast cell numbers (*p*<0.001, **Figure 2C and 2D–F**). Similarly to what was shown with *N. sativa*, no significant changes in total plasma IgE and OVA-specific IgE were observed and intragastric treatment of mice with thymoquinone did not cause any modulation in IL-4, IL-5, IL- 10 or IFNγ secretion by mesenteric lymphocytes after *ex vivo* re-stimulation with OVA.

### Thymoquinone displaces OR-ligands *in vitro*

Thymoquinone has been suggested to activate ORs. To investigate whether ORs play a role mediating the positive effects of thymoquinone and *N. sativa* in OVA-induced allergic diarrhea, we first tested possible interactions of thymoquinone with ORs *in vitro*. At a concentration of 100 µM thymoquinone showed displacement of specific OR-ligands from their receptors in a radio-ligand displacement assay. 45% of [^3^H]DAMGO were displaced from MOR, 35% of [^3^H]U-69,593 from KOR, and 48% of [^3^H]DADLE from DOR, indicating possible binding and subsequent signalling of thymoquinone through OR (**Table 1**).

**Table 1:** *In vitro* ligand displacement by thymoquinone.

Opioid receptor	% ligand displacement with 100 µM Thymoquinonea)
MOR	45
KOR	35
DOR	48

a) 20–50% = moderate displacement (Abbr.: MOR = μ-opioid receptor, KOR = ê-opioid receptor, DOR = δ-opioid receptor).

### ORs are involved in mediating the positive effect of thymoquinone and *N. sativa*

We next investigated the effect of OR-antagonist pre-treatment on thymoquinone induced alleviation of allergic diarrhea in mice. Subcutaneous pre-treatment of mice with the peripheral OR-antagonist, naloxone-methiodide (30 mg/kg BW), strongly reduced the positive effect of thymoquinone on the sum of clinical scores in OVA-allergic mice (*p* = 0.01, **Figure 2A**) as well as the thymoquinone-induced decrease in plasma MMCP-1 (*p* = 0.02, **Figure 2B**) and intestinal mast cell numbers (*p* = 0.001, **Figure 2C and 2G**).

Pre-treatment with 10 mg/kg BW of naloxone-methiodide also reversed the protective effect of *N. sativa* seed extract (**Figure 1A**) whereas, surprisingly, 30 mg/kg did not (**Figure 1B**). Pre-treatment with Naloxone-methiodide did not have any impact on *N. sativa* mediated decrease of MMCP-1 and intestinal mastocytosis (**Figure 1C** **+D**). Naloxone-methiodide alone showed no effect at 10 mg/kg BW and a mild effect when given at 30 mg/kg BW when compared to positive control groups (**Figures 1A and 1B**).

### MOR- and KOR are involved in allergic diarrhea

**Figure 3:**
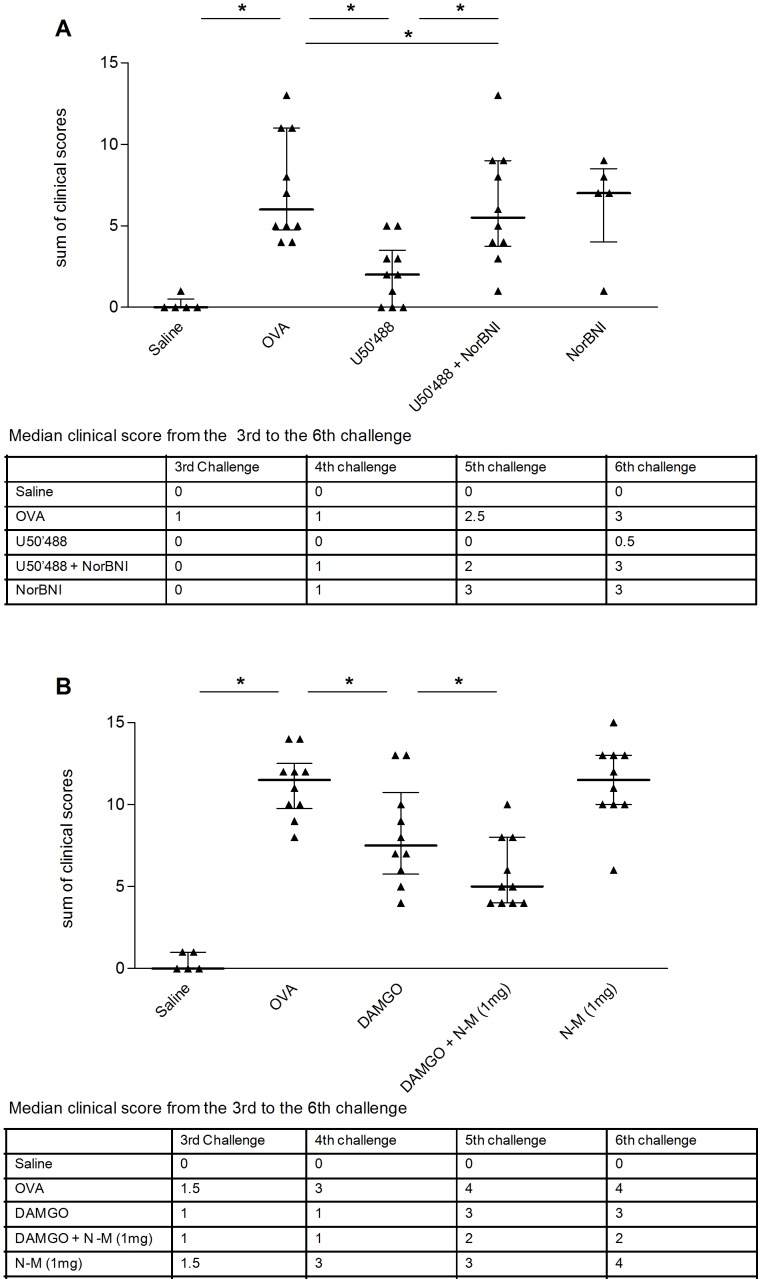
KOR-agonist (U50'488) and MOR-agonist (DAMGO) decrease symptoms in OVA-allergic mice. OVA-sensitized mice were challenged with saline (Saline) or OVA (OVA) with or without pre-treatment. Panel **A** shows the sum of clinical symptoms after subcutaneous treatment with 5 mg/kgBW KOR-agonist U50'488 (U50'488) and 20 mg/kgBW NorBNI (U50'488+NorBNI), or with NorBNI alone (NorBNI). Panel **B** shows the sum of clinical symptoms after subcutaneous treatment with 5 mg/kgBW MOR-agonist (DAMGO) and naloxone-methiodide (DAMGO+N-M 1 mg), or naloxone-methiodide alone (N-M 1 mg). Each dot represents the corresponding value for one animal and the bars represent the median with interquartile range, * = *p*<0.05; n = 5–10. For both panels the table shows the median clinical score for each challenge from the 3^rd^ to 6^th^ challenge.

Since the role of ORs in OVA-induced allergic diarrhea of mice has not been demonstrated previously, we next assessed the influence of OR activation on clinical symptoms and immune parameters, using known OR-agonists. Involvement of DOR, MOR and KOR was tested using the chemical agonists SNC80 and DPDPE, DAMGO, and U50'488, respectively. No measurable effect was observed using the DOR-agonist but pre-treating mice by subcutaneous injection with the KOR-agonist U50'488 at a concentration of 5 mg/kg BW, resulted in a significant, reproducible decrease of the sum of clinical scores (*p*<0.001), due to continuously lower scores from the 3^rd^ challenge onwards (**Figure 3A**), and a low clinical macroscopic diarrhea scores at the last challenge (*p* = 0.005, data not shown). The beneficial effect of U50'488 was strongly reduced in mice pre-treated with the subcutaneous KOR-specific antagonist NorBNI (**Figure 3A**). Administration of NorBNI alone had no effect on diarrhea scores (**Figure 3A**). In order to investigate whether the effect seen with the KOR-agonist U50'488 was purely anti-diarrheic, mice were treated with U50'488 only at the time of diarrhea induction (for two days before the 6^th^ challenge). This short term treatment failed to protect mice from OVA-induced diarrhea.

As with U50'488, subcutaneous injection with the MOR-agonist DAMGO at a concentration of 5 mg/kg BW, resulted in a significant decrease in the sum of clinical scores (*p* = 0.038) due to decreased scores from the 3^rd^ challenge onwards (**Figure 3B**). Subcutaneous injection of OR-antagonist naloxone-methiodide at a concentration of 1 mg/kg BW (a concentration suggested to preferably inhibit MOR) one hour before the administration of DAMGO further attenuated the diarrhea scores, whereas administration of naloxone-methiodide alone showed no effect (**Figure 3B**).

**Figure 4:**
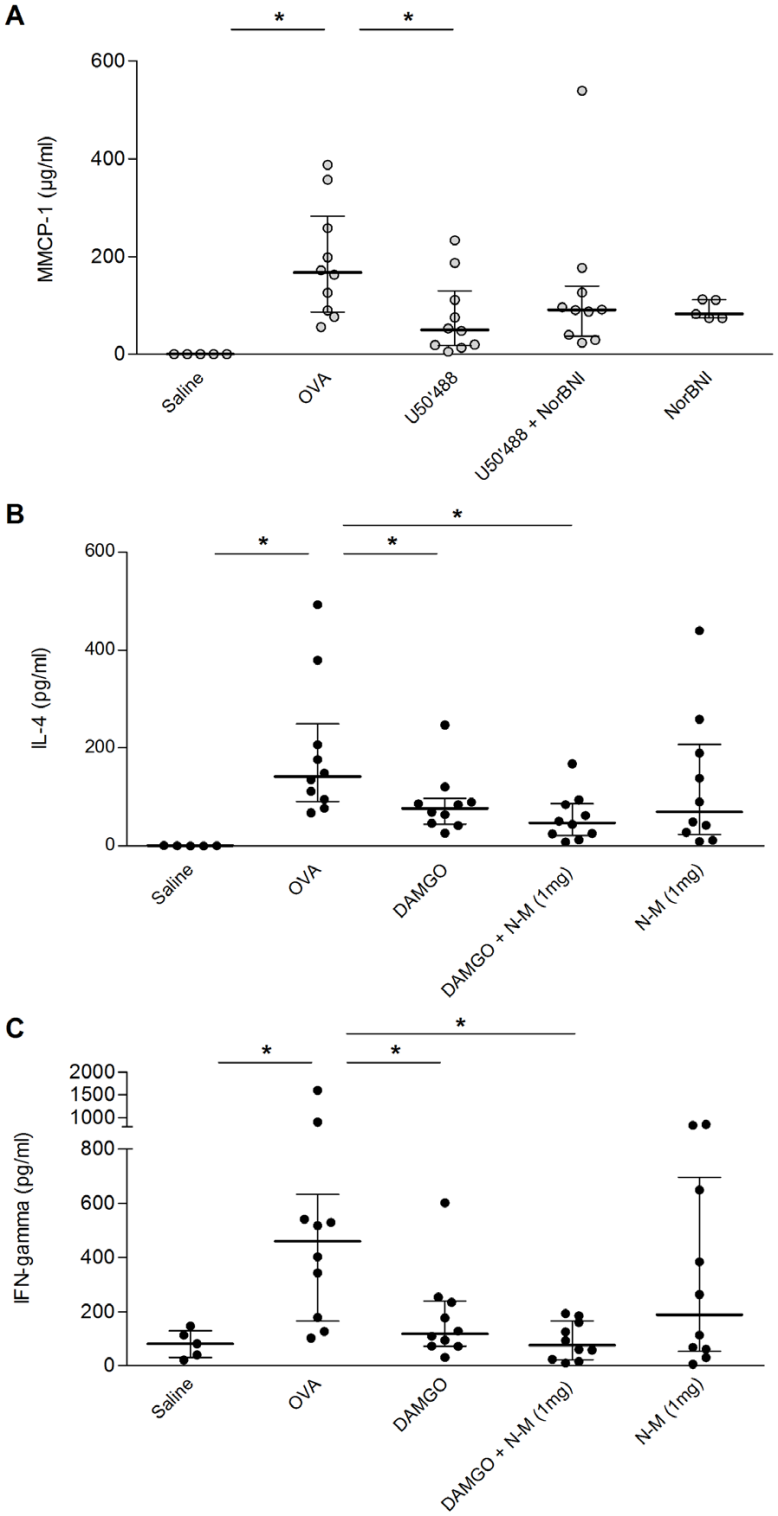
KOR-agonist (U50'488) and MOR-agonist (DAMGO) alleviate allergy related immune markers in OVA-allergic mice. OVA-sensitized mice were challenged with saline (Saline) or OVA (OVA) with or without pre-treatment. The graph shows the concentration of total plasma MMCP-1 (**A**) at sacrifice after subcutaneous treatment with 5 mg/kgBW KOR-agonist U50'488 (U50'488), with 20 mg/kgBW NorBNI (U50'488+NorBNI), or with NorBNI alone (NorBNI), and the concentration of plasma IL-4 (**B**) and IFN-gamma (**C**) after subcutaneous treatment with 5 mg/kgBW MOR-agonist (DAMGO) and naloxone-methiodide (DAMGO+N-M 1 mg), or naloxone-methiodide alone (N-M 1 mg). MMCP-1 levels were unchanged with MOR-agonist and IL-4 and IFN-gamma levels were unchanged with KOR-agonist respectively (data not shown). Each dot represents the corresponding value for one animal and the bars represent the median and interquartile range, * *p*<0.05; n = 5–10.

**Figure 5:**
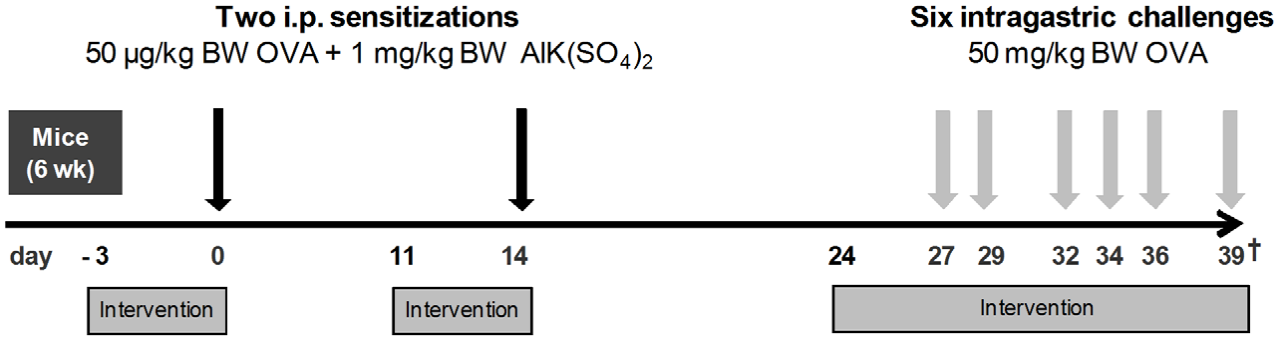
Experimental set up for OVA-induced allergic diarrhea. Intervention corresponds to either intragastric *N. sativa* or thymoquinone (with or without sub-cutaneous OR-antagonist pre-treatment) or subcutaneous OR-agonists (with or without sub-cutaneous OR-antagonist pre-treatment). (Abbreviations: i.p. = intraperitoneal, OR = opioid receptor, OVA = ovalbumin, BW = body weight, † = sacrifice).

**Table 2:** *In vitro* opioid receptor-displacement assay.

Assay used	Receptors/Cells used	Reference compound	Reference of assay	Radio- ligand (concentration)	Incubation
*δ_2_(h)* (DOP)	human recomb./CHO cells	DPDPE	Simonin *et al.* (1994)	[^3^H]DADLE (0.5 nM)	120 min/22°C
κ (KOP)	rat recomb./CHO cells	U 50'488	Meng *et al.* (1993)	[^3^H]U 69'593 (1 nM)	60 min/22°C
*μ(h)* (MOP)	human recomb./HEK-293 cells	DAMGO	Wang *et al.* (1994)	[^3^H]DAMGO (0.5 nM)	120 min/22°C

(Abbr.: CHO = Chinese hamster Ovary, HEK = Human embryonic kidney, DOP = delta opioid; KOP = kappa opioid, MOP = mu opioid, recomb. = recombinant).

### MOR- and KOR-agonists differ in their effect on allergy related immune parameters

KOR-agonist U50'488 decreased the plasma level of MMCP-1 (*p* = 0.01, **Figure 4A**) and total plasma IgE (*p* = 0.043, **Figure S1**). Contrary to the results for clinical symptoms NorBNI pre-treatment did not reverse the effect on MMCP-1 (**Figure 4A**) and only partially reversed the effect on plasma IgE (**Figure S1**). U50'488 showed no significant effect on cytokine release from mesenteric lymphocytes after *ex vivo* stimulation with OVA (data not shown). We next investigated the effect of DAMGO on allergy-related immune parameters. DAMGO had no effect on MMCP-1 and total plasma IgE (data not shown) but decreased the concentration of IL-4 (*p* = 0.02, **Figure 4B**), IFNγ (*p* = 0.02, **Figure 4C**), IL-5 (*p* = 0.02, **Figure S2A**), and IL-10 (*p* = 0.04, **Figure S2B**) after *ex vivo* re-stimulation of mesenteric lymphocytes with OVA. The concentrations of these cytokines were further decreased when mice were pre-treated with naloxone-methiodide whereas naloxone-methiodide alone had no significant effect on cytokine release (**Figure 4B–C** and **Figure S2**).

## Discussion

Here we demonstrate that a hexanic extract of *N. sativa* seeds decreases diarrhea scores and allergy related immune markers in mice with OVA-induced allergic diarrhea. Recent results from the literature indicate beneficial effects of *N. sativa* in respiratory allergies [25], [26]. Detailed studies conducted in *in vivo* allergy models showed a decrease in blood eosinophils, total IgG1, total IgG2 and lung inflammatory cells in mice with allergic asthma [37]. Consistent with these effects, we show that *N. sativa* seed extract also reliably decreased symptoms of food allergy reducing the overall severity of OVA-induced diarrhea in OVA-allergic mice. This effect of *N. sativa* was confirmed in four independent experiments, using two different batches of hexanic *N. sativa* seed extract. The reduction of symptoms is accompanied by a decreased number of OVA-induced intestinal mucosal mast cells and a reduction of OVA-induced plasma MMCP-1 increase. In this mouse model, mast cells have been described as the main mediators of symptoms, which has led to it being considered as a model of local intestinal anaphylaxis [35]. It has been shown that diarrhea is prevented when mast cells were depleted by administration of anti-c-kit antibody or when the effect of mast cell mediators (platelet activating factor and serotonin) was inhibited [35]. Mast cell stabilization and/or decrease in mast cell numbers could therefore be involved in mediating the beneficial effect of *N. sativa* seed extract. Previous data from the literature, regarding *N. sativa* effect on mast cells, substantiate this hypothesis. *In vitro* experiments in rat peritoneal mast cells, pre-treated with nigellone, a carbonyl polymer of thymoquinone (one of the active compounds in *N. sativa*) demonstrated an inhibition of histamine release in response to antigen-specific activation [38]. Furthermore, Kanter and colleagues have reported that protection from gastric ulcers with *N. sativa* treatment in rats was mediated by a decrease in gastric mast cell numbers [39].

In the attempt to also test the influence of *N. sativa* on the adaptive immune response, we re-stimulated murine mesenteric lymph node (MLN) lymphocytes *ex vivo* with OVA to measure cytokine release. Consistent with previously published results [40], cells of *N. sativa* treated mice failed to demonstrate changes in cytokine release. Together these changes suggest that local effector mechanisms rather than changes in sensitization mediate the beneficial effect of *N. sativa* in OVA-allergic mice.

The quinolone, thymoquinone, one of the lipophilic compounds in hexanic extract from *N. sativa* seeds, has been shown to mediate anti-cancer [41], anti-inflammatory [22], and anti-allergic [36] benefits of *N. sativa* in animal models. We have demonstrated with oral administration of thymoquinone to OVA-allergic mice that, similar to the results with *N. sativa* seed extract, thymoquinone provokes a decrease in symptoms, plasma MMCP-1 levels and mast cell numbers in OVA-induced diarrhea. Contrary to previous results in the literature, we did not consistently detect changes in total or OVA-specific IgE after thymoquinone treatment. This discrepancy can most likely be linked to the route of administration (intraperitoneal versus oral). Interestingly, a milk-based extraction of *N. sativa* seeds, containing no thymoquinone, had no effect on allergic diarrhea (data not shown). Together these results suggest that thymoquinone could be a major active component mediating the beneficial effect of *N. sativa* seed extract in OVA-induced allergic diarrhea.

Various mechanisms of action have been suggested for thymoquinone such as inhibition of the cyclooxygenase and 5-lipoxidase pathways [42], and regulation of the toll-like receptor 4/NF êB pathway [43]. It has also been reported that oral thymoquinone as well as *N. sativa* have an analgesic effect in protection from peripheral pain in mice through an OR-mediated mechanism [28]. OR-activation has recently also been reported to have anti-inflammatory properties [44]. We were able to show that thymoquinone displaces OR-agonists *in vitro* suggesting a possible implication of OR-pathways in *N. sativa* and thymoquinone effects. To our knowledge the effects of ORs in food allergy have not been investigated *in vivo*. Thus, we first tested the functional effect of OR-agonists in our mouse model. MOR and KOR are the two ORs that are most widely expressed in the gut, whereas DOR is less abundant. Treatment with either DAMGO (MOR-agonist) or U50'488 (KOR-agonist) significantly improved the severity of OVA-induced allergic diarrhea whereas DOR-agonist showed inconclusive results (data not shown). Peripheral OR-agonists have been demonstrated in the past to delay intestinal transit time and decrease intestinal secretion through neurogenic mechanisms resulting in a beneficial effect on diarrhea [32], [33]. Interestingly, KOR-agonist treatment did not only decrease diarrhea – OR-agonist have long history as anti-diarrhoeic drugs [45], [46] – but also mast cell numbers and plasma MMCP-1 concentration in OVA-allergic mice. This suggests an effect that involves the mucosal immune response. Immune cells, specifically mucosal mast cells have been shown to be in close proximity to neurons [47] in the intestine and they functionally interact [48], [49]. OR could be involved in this interaction since *Ussing* chamber experiments with intestine from beta–lactoglobulin sensitized pigs, revealed that beta–lactoglobulin specific, mast cell induced, neurogenic increase of intestinal secretion is alleviated by OR-agonist treatment [48]. However, future experiments are required to determine the mechanism of the KOR effect. It is noteworthy that KOR-agonist did not show an effect on allergic diarrhea in our mouse model when administered for only two days before the final challenge, reinforcing the suggestion that the benefit of KOR activation in allergic diarrhea goes beyond an anti-diarrheic effect and that a more extended agonist treatment is required to show an effect.

Contrary to KOR-agonist treatment, injection with MOR-agonist DAMGO influenced neither serum MMCP-1 concentration nor mast cell numbers. In line with previous work on MORs [50], which revealed an influence on immune mediator release from inflammatory cells, we observed a significant decrease of cytokines secreted from *ex vivo* OVA-stimulated mesenteric lymphocytes in DAMGO-treated mice. The fact that U50'488 and DAMGO show different immune modulating capacities suggests different mechanisms of action in murine allergic diarrhea but may also result from differences in the expression pattern of KOR and MOR on allergy-related immune cells.

In the experiments with DAMGO we used low dose (1 mg/kg BW) naloxone-methiodide as MOR antagonist, according to its high affinity to MOR. Paradoxically, treatment with low dose DAMGO enhanced the beneficial effect on allergic diarrhea. It has been reported in the literature that MOR-expression on the cell surface is highly regulated and influenced by long term agonist treatment, which can lead to OR-tolerance and opioid dependence. For recycling of functionality following activation, MORs need to be internalized, remodelled, and re-expressed on the surface. Thus one of the mechanisms of agonist tolerance is inactivation of cell surface ORs by circumvention of internalization [51]. On the contrary, low dose, long term antagonist treatment (as used in our model) has been shown to enhance internalization of MOR, resulting in recycling and functional re-expression of the receptor on the cell surface [52]. Combination of high dose agonist and low dose antagonist (like done in our study) could hence, paradoxically increase the total DAMGO signalling through increased availability of functional MOR on the cell surface (functional supersensitivity) and consequently result in an increase of the beneficial effect. This has been reported for low dose OR-antagonist treatment by others [52]. It also needs to be considered that endogenous opioid receptor agonists, like beta-endorphin, could be involved in the regulation of MOR expression and might influence the effect of exogenous MOR agonist [53]. Further investigations measuring the expression of MOR and binding studies using selective MOR-antagonists are needed to fully explain these observations.

When testing the involvement of OR in the beneficial effect of thymoquinone and *N. sativa*, antagonist pre-treatment inhibited the effect of thymoquinone and *N. sativa* on diarrhea, mast cell numbers, and plasma MMCP-1 concentration. We hence conclude that the beneficial effect of *N. sativa* seed extract in allergic diarrhea involves OR-signalling, most possibly through thymoquinone. *N. sativa* seed extract, however, contains other active compounds and we can hence not exclude that non-OR-related pathways are involved. Especially, when keeping in mind that the beneficial effect of *N. sativa* was inhibited by antagonist pre-treatment with moderate dose (10 mg/kg BW) but, contrary to thymoquinone, pre-treatment with high dose of 30 mg/kg BW was not effective. Additional work is required to fully understand the underlying mechanisms.

In conclusion, we have shown for the first time, that MOR- and KOR-signalling are involved in murine allergic diarrhea and that treatment with their respective agonists can decrease symptoms by mechanisms that are going beyond a purely anti-diarrheic effect and that involve modulation of immune parameters associated with food allergy. Furthermore, we were able to show that this pathway might have a clinical application by using an extract from *N. sativa* seeds, which alleviates symptoms of allergic diarrhea and modulates immune parameters in mice. The beneficial effect of *N. sativa* extract seems to be at least partially mediated by its active ingredient thymoquinone and involves OR-signalling. *N. sativa* seed extract might be a promising candidate for nutritional interventions in humans suffering from food allergy.

## Materials and Methods

### Ethics statement

All experiments were approved by the District Veterinary Office of the Canton Vaud, Lausanne, Switzerland and all procedures conducted according to the Swiss Animal Protection Law (Authorization # 1822 VD).

### Animals

Male *BALB/c* mice (Charles River Breeding Laboratories, France) were housed in groups of 5 animals in micro-isolator cages in the Central Animal Facilities at the Nestlé Research Centre, Lausanne, Switzerland. The cages were equipped with filter hoods and kept under controlled temperature (20°C) with a 12∶12 h light-dark cycle and free access to food and water. The animals were given one week after arrival at the animal facility to adapt to the new environment and minimize stress. Afterwards they were randomized by weight into the different experimental groups.

### Sensitization and challenges (OVA-induced allergic diarrhea)

Sensitization and challenge procedures were conducted as previously published [35]. Briefly, the mice (6 weeks old) received two intra-peritoneal injections with 50 µg chicken OVA (Sigma-Aldrich, Germany) and 1 mg aluminium potassium sulphate adjuvant (Alum) (Sigma-Aldrich, Germany) at intervals of two weeks (**Figure 5**). Tail vein blood samples were tested for OVA-specific IgE one week after the second i.p. injection to assess sensitization levels. Thirteen days after the second sensitization the mice were challenged with 50 mg OVA by intra-gastric gavage, placed in individual cages and monitored for diarrhea symptoms during one hour. The mice were scored according to feces appearance as follows: score 0 = no changes; score 1 = soft but well formed; score 2 = soft, non-formed; score 3 = one episode of liquid diarrhea; score 4 = at least two episodes of liquid diarrhea; score 5 (only at sacrifice) = score 4 and only clear liquid in the colon at sacrifice. One hour after the 6^th^ challenge mice were sacrificed by exsanguination under inhalation anaesthesia (isoflurane) and blood, MLN, and intestinal tissue were taken for further analysis.

### Hexanic extraction of *N. sativa* seeds

500 g of *N. sativa* seeds (Mehran Spice & Food Industries, Pakistan) were ground using a laboratory mill. The ground seeds were placed in a flask equipped with an overhead stirrer Heidolph 2041 (Heidolph Instruments GmbH & Co KG, Germany). The oil was extracted twice for 1 hour using hexane (2×2500 ml). The mixture was filtered and the solvent was removed using a rotatory evaporator. The oil was recovered and stored at −20°C until use.

### Treatments

*N. sativa* seed hexanic extract and thymoquinone (Sigma-Aldrich, Germany) were given by intra-gastric gavage once a day for 4 days starting 3 days before the two intraperitoneal sensitizations. Treatment was started again 3 days before the first challenge and continued daily until sacrifice (**Figure 5**). The DOR-agonist 4-[(*R*)-[(2*S*,5*R*)-4-allyl-2,5-dimethylpiperazin-1-yl](3-methoxyphenyl)methyl]-*N, N*-diethylbenzamide (SNC80) or [D-Pen2,5]-Enkephalin (DPDPE), the MOR-agonist [D-Ala^2^, N-MePhe^4^, Gly-ol]-enkephalin (DAMGO), and the KOR-agonist 2-(3,4-dichlorophenyl)-N-methyl-N-[(1R,2R)-2-pyrrolidin-1-ylcyclohexyl]acetamide (U50'488) (all agonists from Sigma-Aldrich, Germany) were injected subcutaneously at a concentration of 5 mg/kg BW once a day. When indicated, mice were injected subcutaneously with the peripheral OR-antagonist naloxone–methiodide (N-M) (concentration of 1 mg/kg, 10 mg/kg or 30 mg/kg) or the KOR-antagonist Norbinaltorphimine (NorBNI) (concentration 20 mg/kg) (both Sigma-Aldrich, Germany) 1 h prior to the agonist treatment. All subcutaneous administered substances were dissolved in sterile saline. Due to the long lasting antagonist effect of NorBNI the injection was given every other day.

### *In vitro* OR-binding assay

Interaction of thymoquinone with OR was tested *in vitro* for MOR, KOR and DOR at CEREP (Le Bois l'Evêque, BP 1, 88660 Celle l'Evescault) using previously published radiolabel-binding assays [54]–[56] with the conditions displayed in **Table 2**. In each of the assays, thymoquinone was tested concurrently with the respective reference compound (MOR-agonist (DAMGO), KOR-agonist (U-50'488) or DOR-agonist (DPDPE)) at several concentrations (10^−6^, 10^−5^, 10^−4^ M). Displacement of radiolabled ligands was measured by scintillation counts. The specific binding was determined as the difference between total binding of the radiolabeled ligands and nonspecific binding, determined in the presence of an excess of unlabelled ligands. Results were displayed in % dislocation of radiolabeled MOR-agonist ([^3^H]DAMGO), KOR-agonist ([^3^H]U-69,593) or DOR-agonist ([^3^H]DADLE). Displacement of 25–50% was rated as moderate displacement and 51–100% as strong displacement.

### *Ex vivo* re-stimulation of mesenteric lymph node

MLN isolated cells were re-stimulated *ex vivo* with OVA as described previously [57]. Briefly, MLNs were homogenized with a syringe plunger in a cell strainer (BD Falcon, Switzerland). Cells were centrifuged and washed 2× in RPMI medium (Sigma) complemented with 10% fetal bovine serum (FBS; Bioconcept, France), 1% L-glutamine, 1% Penicillin/Streptomycin 0.1% Gentamycin, 50 mM β-mercaptoethanol (all: Sigma-Aldrich, Germany). Cells (3×10^5^ cells/well) were cultured in 96 well flat bottom plate (Milian, Switzerland) in the absence or presence of OVA (1 mg/ml). After 72 hrs of culture, plates (including supernatant and cells) were frozen at −20°C.

### Quantification of cytokines in cell culture supernatant

Mouse IL-4, IL-5, IL-10, and IFNγ were measured using the mouse Th-1/Th-2 4-plex multiplex kit (MesoScale Discovery®, USA) according to the manufacturer's instructions.

### Quantification of total plasma IgE and OVA-specific IgE by ELISA

Blood was collected in heparin tubes at sacrifice, centrifuged, aliquoted, and stored at −20°C until use. Total IgE concentration and mouse mast cell protease-1 (MMCP-1) were measured by ELISA Kit (BD Bioscience, USA and Moredune Scientific, UK, respectively) according to the manufacturer's instructions. For detection of OVA-specific IgE [58], 96 well plates were coated with anti-mouse IgE antibody (BD Pharmingen, Switzerland) over night. The next day plates were blocked with 1% bovine serum albumin in phosphate buffered saline plus 0.5% Tween20 (Sigma Aldrich, Germany) (PBSAT) for 1 h at 37°C, washed with PBSAT and samples were added starting with a dilution serum to PBSAT 1∶10 diluting sequentially 1∶2. Mouse anti-OVA IgE (Serotec, UK) served as standard with a starting concentration of 200 ng/ml. Following incubation at 37°C for 2 h, plates were washed and biotinylated OVA (see below) was added at a concentration of 4 µg/ml with a subsequent incubation for 1 h at 37°C. Before development with 3,3′,5,5′ – tetramethylbenzidine (KPL, US) plates were incubated for 30 min in the dark at 37°C with horse radish peroxidase-conjugated streptavidine (KPL, US). Colorimetric reaction was stopped with 1 N HCl.

The ODs of all ELISAs were read at 450 nm with a Dynex MRX II (Dynex Technologies, Germany). For biotinylation, 2 ml of OVA (10 mg/ml) and 1 mg of sulfo-LC-NHS-biotin (Molecular Biosciences, US) were incubated on a rotating wheel for 1 h at RT. The solution was then loaded on a PD-10 Sephadex G25M column (Amersham Biosciences, Sweden) to remove excess biotin. Protein concentration was determined using standard procedures and biotinylation was verified by Western Blot analysis.

### Mast cell quantification

For mast cell quantification in the small intestinal mucosa 0.5 cm of the proximal jejunum were rinsed with PBS, placed in 10% formalin (Sigma-Aldrich, Germany), and embedded in paraffin using standard histological techniques. Slides (5 µm) were cut and mucosal mast cells stained for chloroacetate esterase activity [35] and tissue counter stained with haematoxylin. Mast cell numbers were determined by counting chloroacetate-positive cells under a light microscope (400× magnification). Mast cells in at least 5 villi (OVA-challenged groups) or 10 villi (saline-challenged groups) were counted and the results were expressed in number of mast cells/villus.

### Statistics

For each experiment a gate keeper strategy was used, that allowed comparisons between treatment groups and OVA-challenged positive controls only if the groups “saline” and “OVA” showed significant differences. Results are expressed as median with interquartile ranges. Clinical macroscopic scores at the 6^th^ challenge comprised clinical and *in situ* observations (scores 1–5). Clinical sum of scores was calculated as sum of the clinical scores (scores 1–4) from all 6 challenges with a score of 0 assigned to the 1^st^ and 2^nd^ challenge according to the progressive nature of the symptoms with multiple challenges. Wilcoxon-Test was used to analyze differences between groups. Tests were performed two-sided. *P*-values of less than 0.05 were considered significant. The software used for the calculations was R 2.6.1 (http://www.r-project.org).

## Supporting Information

Figure S1 **KOR-agonist (U50'488) decreases total plasma IgE in OVA-allergic mice.** OVA-sensitized mice were challenged with saline (Saline) or OVA (OVA) with or without pre-treatment. The graph shows the concentration of plasma IgE at sacrifice after subcutaneous treatment with 5 mg/kgBW KOR-agonist U50'488 (U50'488), with 20 mg/kgBW NorBNI (U50'488+NorBNI), or with NorBNI alone (NorBNI). Each dot represents the corresponding value for one animal and the bars represent the median and interquartile range, * *p*<0.05; n = 5–10.
(TIF)

Figure S2 **MOR-agonist (DAMGO) decreases IL-5 and IL-10 in OVA-allergic mice.** OVA-sensitized mice were challenged with saline (Saline) or OVA (OVA) with or without pre-treatment. Panels **A** and **B** show the concentration of IL-5 (**A**) and IL-10 (**B**) in supernatant from *ex vivo* re-stimulated mesenteric lymphocytes of mice subcutaneously treated with 5 mg/kgBW MOR-agonist (DAMGO) and naloxone-methiodide (DAMGO+N-M 1 mg), or naloxone methiodide alone (N-M 1 mg). Each dot represents the corresponding value for one animal and the bars represent the median and interquartile range, * *p*<0.05; n = 5–10.
(TIF)
