# Supplementary figures and images for: Nigella sativa (Black Cumin) Seed Extract Alleviates Symptoms of Allergic Diarrhea in Mice, Involving Opioid Receptors
Source: PLoS One. 2012 Jun 29;7(6):e39841. doi: 10.1371/journal.pone.0039841 (PMC3387213; doi:10.1371/journal.pone.0039841)

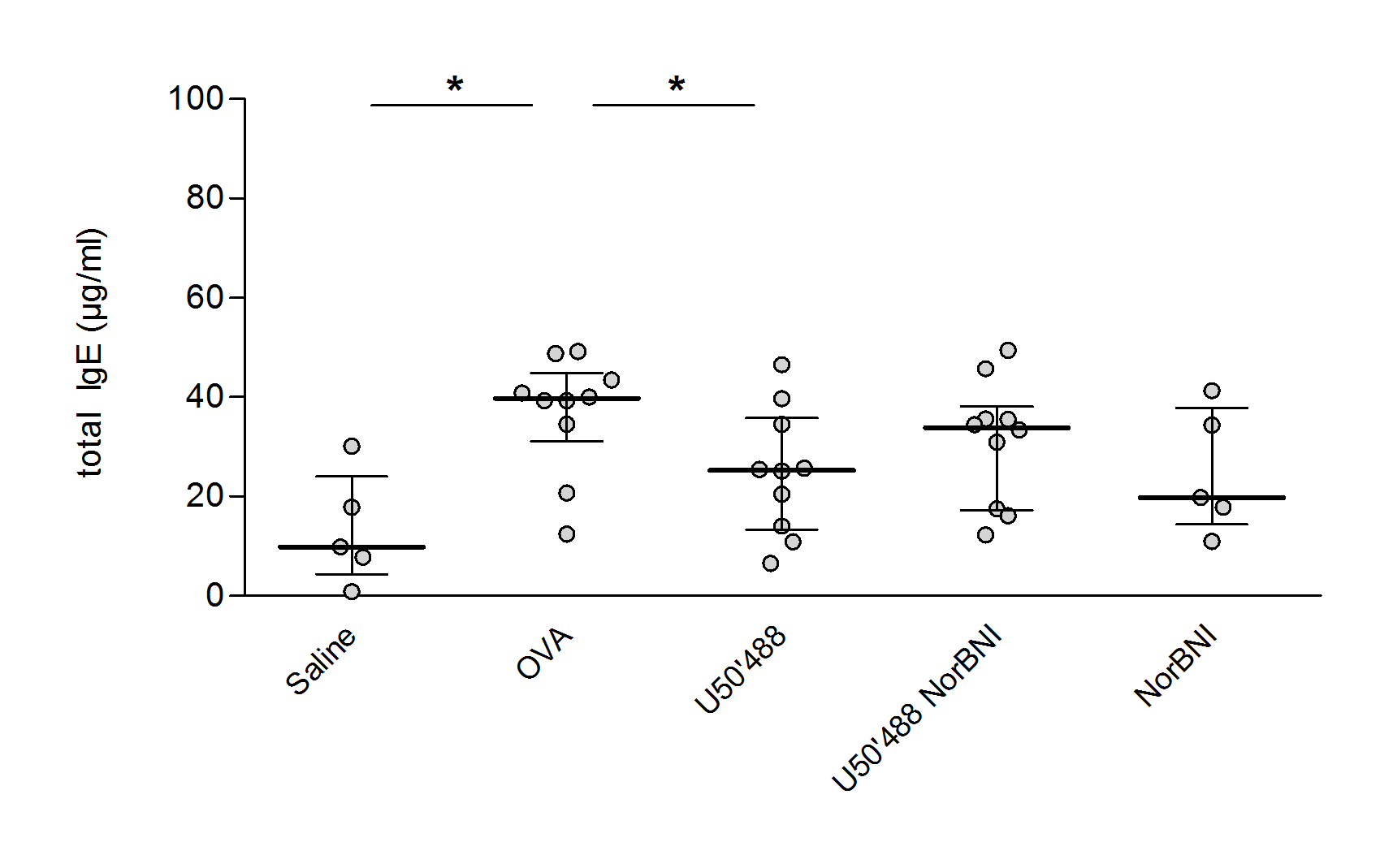

Supplement: Figure S1 — KOR-agonist (U50'488) decreases total plasma IgE in OVA-allergic mice. OVA-sensitized mice were challenged with saline (Saline) or OVA (OVA) with or without pre-treatment. The graph shows the concentration of plasma IgE at sacrifice after subcutaneous treatment with 5 mg/kgBW KOR-agonist U50'488 (U50'488), with 20 mg/kgBW NorBNI (U50'488+NorBNI), or with NorBNI alone (NorBNI). Each dot represents the corresponding value for one animal and the bars represent the median and interquartile range, * p<0.05; n = 5–10. (TIF) [file pone.0039841.s001.tif]

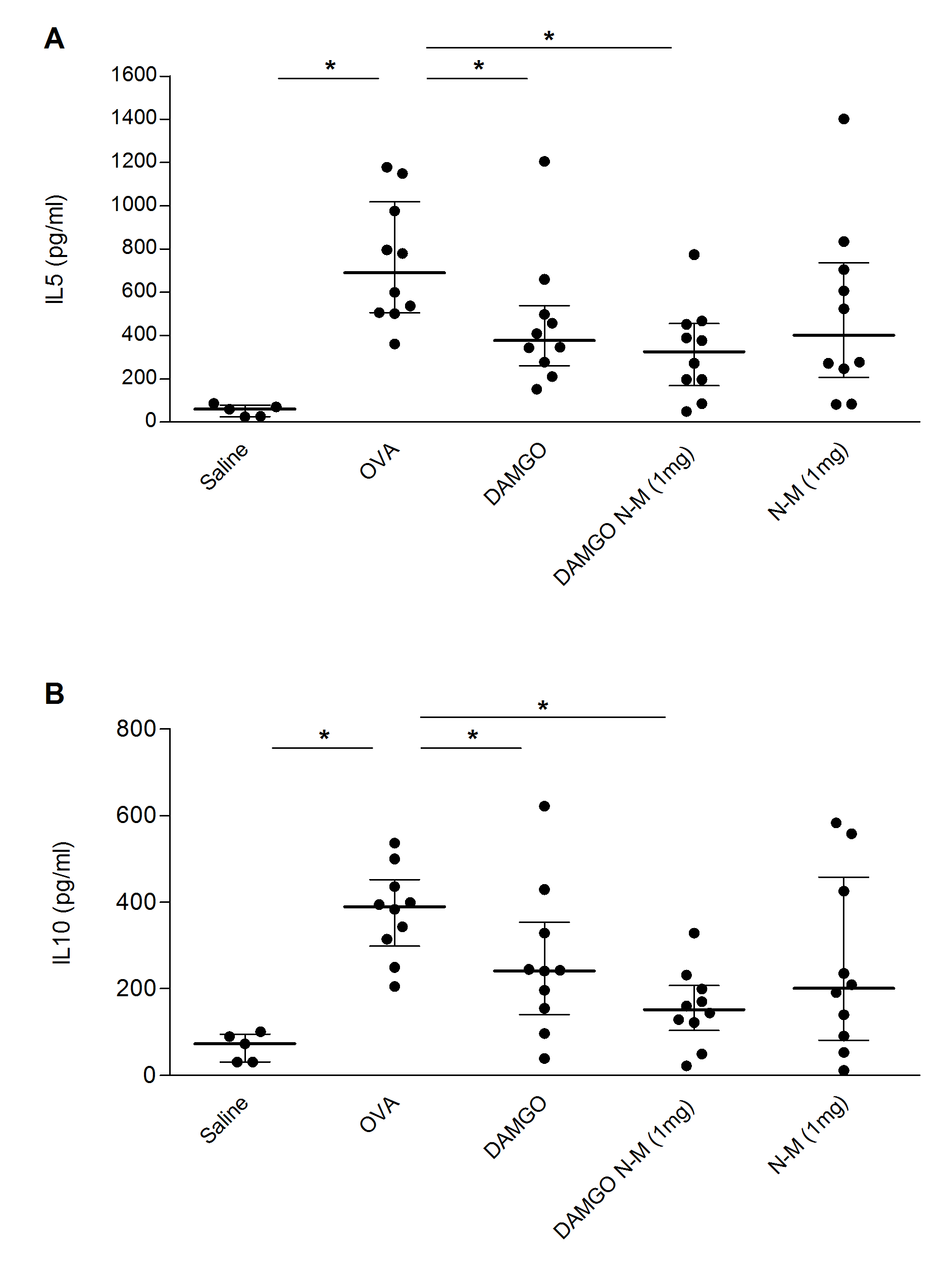

Supplement: Figure S2 — MOR-agonist (DAMGO) decreases IL-5 and IL-10 in OVA-allergic mice. OVA-sensitized mice were challenged with saline (Saline) or OVA (OVA) with or without pre-treatment. Panels A and B show the concentration of IL-5 (A) and IL-10 (B) in supernatant from ex vivo re-stimulated mesenteric lymphocytes of mice subcutaneously treated with 5 mg/kgBW MOR-agonist (DAMGO) and naloxone-methiodide (DAMGO+N-M 1 mg), or naloxone methiodide alone (N-M 1 mg). Each dot represents the corresponding value for one animal and the bars represent the median and interquartile range, * p<0.05; n = 5–10. (TIF) [file pone.0039841.s002.tif]
